# Human and zebrafish mineralocorticoid receptors reporter cell assays to assess the activity of chemicals

**DOI:** 10.1016/j.isci.2025.113764

**Published:** 2025-10-14

**Authors:** Anna Toso, Abdelhay Boulahtouf, Marina Grimaldi, Audrey Sansaloni, Yoshinao Katsu, Michael E. Baker, Aurélie Escande, Clémentine Garoche, Selim Aït-Aïssa, Patrick Balaguer

**Affiliations:** 1 Institut de Recherche en Cancérologie de Montpellier (IRCM), Inserm, U1194, Université de Montpellier, Institut régional du Cancer de Montpellier (ICM), 34090 Montpellier, France; 2 Department Environmental Toxicology, Swiss Federal Institute of Aquatic Science and Technology, Eawag, 8600 Dübendorf, Switzerland; 3 Graduate School of Life Science, Hokkaido University, Sapporo 060-0808, Japan; 4 Faculty of Science, Hokkaido University, Sapporo 060-0808, Japan; 5 Division of Nephrology, Department of Medicine, University of California, San Diego, San Diego, CA 92093, USA; 6 Center for Academic Research and Training in Anthropogeny (CARTA), University of California, San Diego, San Diego, CA 92093, USA; 7 UMR Hydrosciences Montpellier, Université de Montpellier, Montpellier, France; 8 Institut National de l'Environnement Industriel et des Risques (INERIS), Unité Écotoxicologie des Substances et Milieux, 60550 Verneuil-en-Halatte, France

**Keywords:** Natural sciences, Environmental monitoring, Biological sciences, Biochemistry, Cell biology

## Abstract

The action of environmental chemicals (ECs) on the mineralocorticoid receptor (MR) has been suggested to impair physiological processes regulated by this nuclear receptor. However, it remains understudied both as a target of ECs and with respect to potential species-specific differences. In this regard, we have developed reporter cell lines to identify the response to different steroids, ECs, and urban wastewater (WW) sample extracts of human MR (hMR) and zebrafish MR (zfMR). Most of the steroids had a higher efficacy on zfMR than hMR, while the ECs were antagonists to both hMR and zfMR, with a lower potency on the latter. Interestingly, WW sample extracts revealed the presence of MR activity with a greater activity on zfMR compared to hMR, suggesting the presence of steroids in WW. These screening tools have proven to be powerful tools for characterizing the interaction of chemicals with MRs and revealing their presence in environmental samples.

## Highlights

• Manifold steroids had a higher efficacy on zfMR than hMR
• This difference in efficacy is due to a substitution of a threonine (hMR) by a leucine (zfMR)
• Environmental chemicals antagonize hMR and zfMR with a lower potency on zfMR
• Waste water revealed the presence of MR activity likely due to steroids

## Graphical abstract

**Figure:**
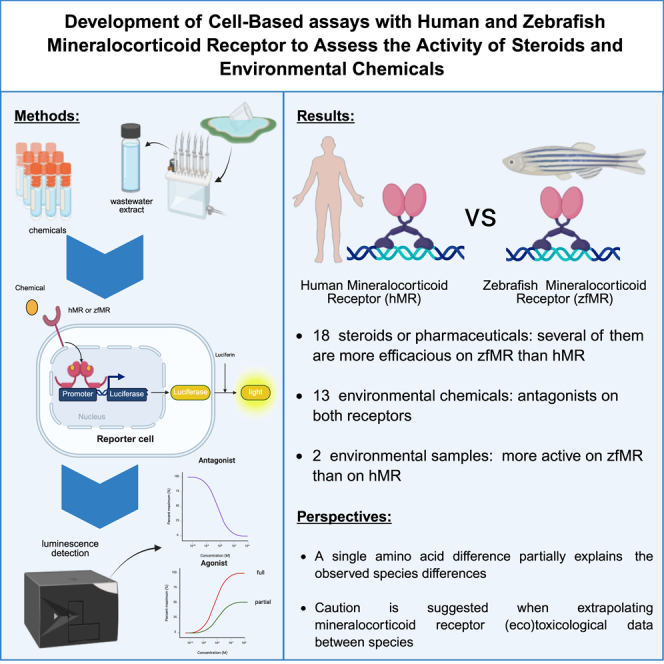

## Introduction

The increasing prevalence of environmental chemicals (ECs), such as pesticides, pharmaceuticals, and industrial compounds, has raised concerns about their endocrine disrupting adverse effects on humans and wildlife. Most of the studies addressing the receptor-mediated endocrine activity of ECs have focused on the ability of these chemicals to interact with estrogen receptors ERα (NR3A1) and ERβ (NR3A2) and androgen receptor AR (NR3C4), both belonging to the NR subfamily of steroid receptors.^1^^,^^2^^,^^3^^,^^4^ Only recently, it has been suggested that such compounds are able to interfere with other members of the same subfamily, such as the progesterone receptor PR (NR3C3), the glucocorticoid receptor GR (NR3C1), and the mineralocorticoid receptor MR (NR3C2).^2^^,^^5^^,^^6^^,^^7^^,^^8^ However, more studies are needed to identify active ECs and better assess their effects on these understudied receptors.

Like other steroid receptors, MR contains an A/B domain in the N-terminus, a DNA-binding domain (C domain) in the center, a hinge domain (DBD) (D domain), and a ligand-binding domain (LBD) (E domain) at the C-terminus. The A/B domain contains a constitutive activation function domain 1, and the E domain contains an AF2 domain, which in the presence of agonistic ligands recruits co-activators.^9^^,^^10^ MR is thus a ligand-activated transcription factor whose main physiological role in humans is to mediate the effects of mineralocorticoids on a variety of target tissues such as the kidney, colon, adipose tissue, cardiovascular and nervous system, and to regulate the Na^+^/K^+^ homeostasis, cell proliferation, and blood pressure.^11^ Given the importance of MR in many physiological pathways, it has been reported that the disruption of MR-regulated pathways may contribute to the onset of cardiovascular diseases, metabolic, and stress-associated disorders.^12^^,^^13^^,^^14^ MR, like the other steroid receptors, is activated upon ligand binding and then translocated to the nucleus, where it recognizes specific palindromic DNA sequences and dimerizes to initiate transcription.

Reporter cell lines (cell lines stably expressing a reporter gene under the transcriptional control of an NR) are powerful *in vitro* screening tools that provide useful information on the functional effects of ligands on steroid receptors. However, the reporter cell lines available so far predominantly express human steroid receptors, which may be of concern when assessing a hazard for aquatic species. It has been recently reported by several research groups, including ours, that there are species differences in the interaction of chemicals with steroid receptors.^15^^,^^16^^,^^17^^,^^18^^,^^19^^,^^20^ Interestingly, it is suggested that MR evolved differently in fish and other vertebrates in order to respond to different steroids. In particular, a leucine at position 856 in helix 8 of the ligand-binding domain of the zebrafish MR (zfMR) (corresponding to threonine 870 in human MR) has been identified as playing an important role in the differential response of zfMR and hMR to the progestin progesterone.^16^

Overall, there is a need to expand our knowledge on the interactions of environmental chemicals of different chemical classes with hMR and zfMR, to highlight possible species-specific differences between human and fish MRs, and to assess the presence of active MR ligands in environmental samples. In this regard, the aim of this study was to develop stable and selective reporter cell lines for hMR and zfMR (UG5LN-GAL4-hMR and UG5LN-GAL4-zfMR, respectively). These new cell lines were first characterized in response to four reference steroidal ligands and then used to screen *in vitro* hMR and zfMR activity of a panel of different chemicals, including natural and synthetic steroids, environmental chemicals, and environmental samples (i.e., wastewater samples).

## Results

### Characterization of the UG5LN, UG5LN-GAL4-hMR, and UG5LN-GAL4-zfMR cell lines in response to reference MR and GR ligands

**Figure 1:**
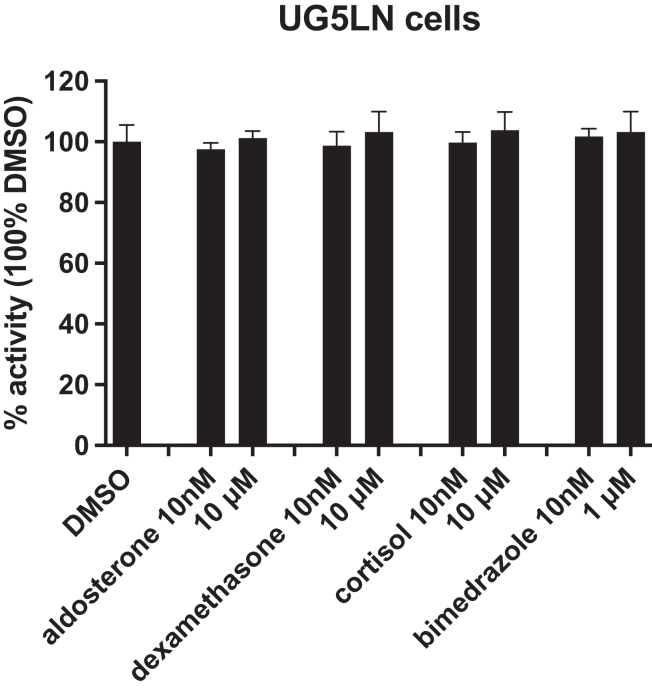
Aldosterone, dexamethasone, bimedrazole, and cortisol do not modulate luciferase expression in the UG5LN cell line The cells were treated with steroids at 10^−8^ M and 10^−5^ M except for bimedrazol, which was used at 10^−8^ M and 10^−6^ M. Results are expressed as the percentage of the maximum luciferase activity in presence of DMSO. Data are presented as means ± SD values. n (replicates per experiment) = 4 per group.

In order to establish an MR reporter cell line, we selected the U2OS cell line, as it expresses low levels of steroid receptors, such as GR, which could have interfered with the results, as many steroids are agonists of both GR and MR. To investigate whether hMR and zfMR are similarly targeted by chemicals, we selected 18 natural and synthetic steroids and 13 ECs known or their anti-androgenic activity (Tables 1 and 2). We firstly tested the effect of aldosterone, cortisol, and dexamethasone, considered as agonists of MR and GR, as well as bimedrazole, a selective agonist of GR^21^^,^^22^ in the UG5LN cell line that was transfected only by the luciferase gene. At 10^−8^ M and 10^−5^ M, none of these four chemicals induced or inhibited luciferase expression (Figure 1), confirming that endogenous NRs in U2OS cells cannot bind and activate/inhibit the (GAL4RE)_5_βGlob-Luc-SVNeo reporter gene.

**Table 1:** Classification of natural and synthetic steroids tested on hMR and zfMR reporter cells

Classification	Compound	Molecular weight	CAS number	Molecular formula	Chemical structure
mineralocorticoids	aldosterone	360.44	52-39-1	C_21_H_28_O_5_	
	drospirenone	366.493	67392-87-4	C_24_H_30_O_3_	
	spironolactone	416.58	52-01-7	C_24_H_32_O_4_S	
	canrenone	340.45	976-71-6	C_22_H_28_O_3_	
	finerenone	378.43	1050477-31-0	C_21_H_22_N_4_O_3_	
	eplerenone	414.49	107724-20-9	C_24_H_30_O_6_	
glucocorticoids	bimedrazole	488.62	4906-84-7	C_30_H_36_N_2_O_4_	
	cortisol	362.46	50-23-7	C_21_H_30_O_5_	
	dexamethasone	392.47	50-02-2	C_22_H_29_FO_5_	
	medrol	374.47	83-43-2	C_22_H_30_O_5_	
	11-deoxycortisol	362.46	152-58-9	C_21_H_30_O_5_	
progestins	progesterone	314.46	57-83-0	C_21_H_30_O_2_	
	dihydroprogesterone	332.48	1662-06-2	C_21_H_32_O_3_	
	promegestone	326.48	34184-77-5	C_22_H_30_O_2_	
	17α-hydroxyprogesterone	330.46	68-96-2	C_21_H_30_O_3_	
	pregnenolone	316.48	145-13-1	C_21_H_32_O_2_	
	mifepristone	429.604	84371-65-3	C_29_H_35_NO_2_	
androgens	dihydrotestosterone	290.44	521-18-6	C_19_H_30_O_2_

**Table 2:** Classification of environmental chemicals tested on hMR and zfMR reporter cells

Classification	Compound	Molecular weight	CAS number	Molecular formula	Chemical structure
Industrial chemicals	Nonylphenol mixture	220.35	284-325-5	C_15_H_24_O	
	4-*tert*-octylphenol	206.32	140-66-9	C_14_H_22_O	
	Bisphenol A	228.29	80-05-7	C_15_H_16_O_2_	
	2-chloro-bisphenol A	262.14	74129-35-1	CH_15_H_15_O_2_Cl	
	Bisphenol C	281.13	14868-03-2	C_14_H_10_Cl_2_O_2_	
Pesticides	aldrin	364.91	206-215-8	C_12_H_8_Cl_6_	
	chlordecone	490.64	143-50-0	C_10_Cl_10_O	
	endosulfan	406.93	115-29-7	C_19_H_6_Cl_6_O_3_S	
	transnonachlore	444.2	39765-80-5	C_10_H_5_Cl_9_	
	2,4′DDE	326.48	3424-82-6	C_22_H_30_O_2_	
	HPTE	317.59	2971-36-0	C_14_H_11_Cl_3_O_2_	
	Vinclozolin M2	260.12	83792-61-4	C_11_H_11_Cl_2_NO_2_

**Figure 2:**
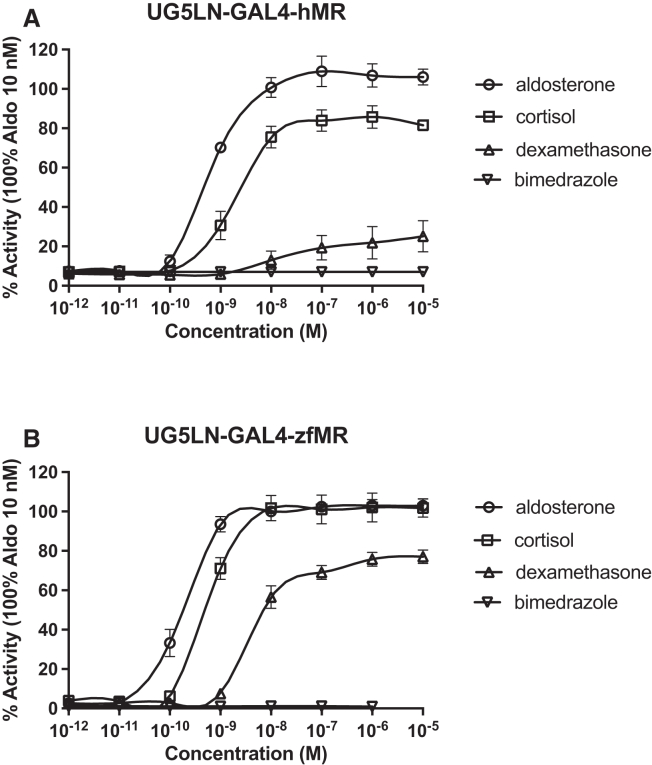
Cortisol and dexamethasone had an higherpotency and efficacy on zfMR than on hMR Dose-response curves of aldosterone, dexamethasone, cortisol, and bimedrazole in UG5LN-GAL4-hMR (A) and UG5LN-GAL4-zfMR cells (B). Results are expressed as the percentage of the maximum luciferase activity induced by 10^−8^ M aldosterone. The curves are presented as a non-linear regression; log (ligand) versus response. EC_50_ values (10^−9^ M) are shown in Tables 3 and 4. Data are presented as means ± SD values. n (replicates per experiment) = 4 per group.

**Figure 3:**
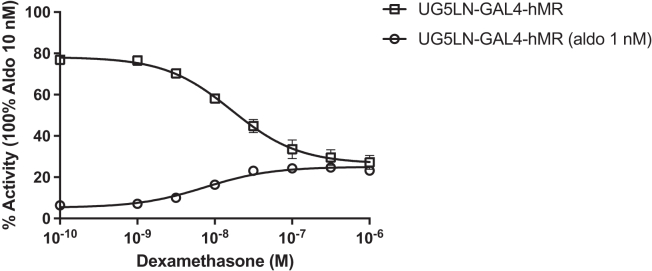
Dose-response curves of dexamethasone in the absence or presence of aldosterone 10^−9^ M in HG5LN-GAL4-hMR cells Results are expressed as the percentage of the maximum luciferase activity induced by 10^−8^ M aldosterone. The curves are presented as a non-linear regression; log (ligand) versus response. EC_50_ and IC_50_ values (10^−9^ M) are shown in Table 3. Data are presented as means ± SD values. n (replicates per experiment) = 4 per group.

In the UG5LN-GAL4-hMR cells (Figure 2A), which expressed the ligand binding domain of hMR fused with the DNA binding domain of yeast GAL4, aldosterone was as a full agonist with an EC_50_ of 0.7 10^−9^ M. Cortisol was an agonist with a maximum activity of 85.8% and an EC_50_ of 3.5 10^−9^ M (Table 3), while dexamethasone only partially activated luciferase expression, with a maximum activity of 25.1% and an EC_50_ of 8.2 10^−9^ M. In co-exposure with aldosterone 10^−9^ M (i.e., antagonistic mode), dexamethasone partially reduced luciferase expression, confirming its partial agonism on hMR with an IC_50_ of 19.9 10^−9^ M (Figure 3; Table 3). Finally, bimedrazole was not active on its own (Figure 2A). Moreover, in the presence of 10^−9^ M aldosterone, bimedrazole was not able to decrease luciferase expression, confirming the absence of interaction of this chemical with hMR (data not shown).

**Table 3:** Agonistic and antagonistic hMR activity assessment

Ligands	EC50 (10^−9^M)	lower and upper 95% conf. limit (10^−9^M)	% max act	REP	IC50 (10^−9^M)	lower and upper 95% conf. limit (10^−9^M)	% min act
DMSO	–	–	7.1 ± 1.1	–	–	–	–
aldosterone	0.69	0.64 to 0.75	100 ± 5	1	–	–	–
drospirenone	–	–	–	–	2.71	2.41 to 3.07	6.9 ± 1.3
spironolactone	–	–	–	–	9.89	8.64 to 11.28	7.3 ± 2.1
canrenone	–	–	–	–	16.7	15.1 to 18.4	7 ± 0.5
finerenone	–	–	–	–	2.43	2.1 to 2.79	11 ± 0.6
eplerenone	–	–	–	–	152	123 to 187	11 ± 3
bimedrazole	NA	–	–	–	NA	–	–
cortisol	3.46	2.9 to 4.15	85.8 ± 5.8	0.2	–	–	–
dexamethasone	8.2	7.28 to 9.5	25.1 ± 7.9	0.08	19.9	16.7 to 23.8	27.2 ± 3.3
medrol	4.1	3.1 to 5.5	47.8 ± 3.9	0.17	12.9	10.1 to 16.4	42.5 ± 1.1
11-deoxycortisol	–	–	–	–	4.41	2.51 to 9.25	11 ± 2.1
progesterone	–	–	–	–	6.43	3.77 to 10.97	9.6 ± 1.4
dihydroprogesterone	–	–	–	–	395	293 to 520	11 ± 2
promegestone	–	–	–	–	169	153 to 186	6.0 ± 1.2
17α-hydroxy progesterone	0.89	0.42 to 1.88	43.5 ± 0.9	0.78	3.95	2.09 to 7.46	50.8 ± 8.3
pregnenolone	–	–	–	–	67.7	47.5 to 96.5	6.0 ± 1
mifepristone	–	–	–	–	836	633 to 1081	4.3 ± 0.4
dihydrotestosterone	–	–	–	–	60	40.4 to 79.7	6.1 ± 1.1

Half maximal effective concentration (EC_50_) and maximal activity (% max act) of the agonistic chemicals, half maximal inhibitory concentration (IC_50_) and minimal activity of the antagonistic chemicals on hMR.

EC_50_s and IC_50_s are expressed in nanomolar (10^−9^ M). Values of EC_50_ and IC_50_s are the mean from at least three separate experiments. Lower and upper 95% confidence limits of EC_50_s and IC_50_s are indicated. Maximal activities (% max act) of the chemicals tested for their agonistic activity and minimal activities (% min act) of the chemicals tested for their antagonistic activity are expressed as a percentage of the maximal luciferase activity induced by 10^−8^ M aldosterone. They were determined at 10^−5^ M for aldosterone, canrenone, cortisol, dexamethasone, promegestone, mifepristone, and dihydrotestoterone, at 10^−6^ M for bimedrazole, spironolactone, and medrol, and at 10^−7^ M for drospirenone and 17α-hydroxy progesterone. Relative potency (REP) of each agonistic chemical was calculated as a ratio of concentrations of aldosterone or chemical required to induce the specific transactivation by 50% (ratio of EC50 values). REP value for aldosterone was arbitrarily set at 1. NA. Non active.

To investigate potential differences between hMR and zfMR, we established a similar reporter gene cell line for zfMR (i.e., the UG5LN-GAL4-zfMR cell line). We then tested the effect of aldosterone, cortisol, dexamethasone and bimedrazole on luciferase expression in these cells (Figure 2B). Both aldosterone and cortisol were full zfMR agonists in UG5LN-GAL4-zfMR with EC_50s_ of 0.30 and 0.87 10^−9^ M, respectively (Table 4). Dexamethasone was an agonist with a maximal activity of 76% and an EC_50_ of 2.7 10^−9^ M (Table 4). Finally, bimedrazole was unable to modulate luciferase expression alone (Figure 2B) or in the presence of 0.5 10^−9^ M aldosterone (data not shown), indicating that this chemical does not bind to zfMR. Interestingly, aldosterone was slightly more potent on zfMR than hMR, while cortisol and dexamethasone clearly had a higher potency and efficacy on zfMR than on hMR.

**Table 4:** Agonistic and antagonistic zfMR activity assessment

Ligands	EC50 (10^−9^M)	lower and upper 95% conf. limit (10^−9^M)	% max act	REP	IC50 (10^−9^M)	lower and upper 95% conf. limit (10^−9^M)	% min act
DMSO	–	–	2.6 ± 0.8	–	–	–	–
aldosterone	0.30	0.26 to 0.36	100 ± 1.4	1	–	–	–
drospirenone	1.68	1.25 to 2.30	57.0 ± 5.8	0.18	2.08	0.89 to 5.10	55.6 ± 1.4
spironolactone	7.29	6.28 to 8.47	35.3 ± 3.1	0.04	5.56	4.91 to 6.31	38.3 ± 1.4
canrenone	–	–	–	–	18.4	15.3 to 22.3	4.0 ± 0.6
finerenone	–	–	–	–	40.4	39.7 to 41.4	3 ± 0.4
eplerenone	–	–	–	–	87.8	51.5 to 133	5 ± 0.3
bimedrazole	NA	–	–	–	NA	–	–
cortisol	0.87	0.66 to 1.14	101.1 ± 4.6	0.34	–	–	–
dexamethasone	2.69	2.55 to 2.83	75.8 ± 3.5	0.11	–	–	–
medrol	1.77	0.92 to 2.50	101.3 ± 2.5	0.17	–	–	–
11-deoxycortisol	0.73	0.64 to 0.84	74 ± 2	0.41	–	–	–
progesterone	2.31	1.26 to 4.20	66.2 ± 2.4	0.13	3.25	1.46 to 7.25	67.0 ± 1.1
dihydroprogesterone	–	–	–	–	186	181 to 189	5 ± 0.5
promegestone	–	–	–	–	291	254 to 332	7.0 ± 1.3
17-α-hydroxy progesterone	0.24	0.12 to 0.46	97.6 ± 1.2	1.25	–	–	–
pregnenolone	–	–	–	–	166	104 to 264	4.1 ± 0.2
mifepristone	–	–	–	–	1055	835 to 1290	3.0 ± 0.6
dihydrotestosterone	–	–	–	–	34.9	24.1 to 48.3	4.2 ± 0.5

Half maximal effective concentration (EC_50_) and maximal activity (% max act) of the agonistic chemicals, half maximal inhibitory concentration (IC_50_) and minimal activity of the antagonistic chemicals on zfMR.

EC_50_s and IC_50_s are expressed in nanomolar (10^−9^ M). Values of EC_50_ and IC_50_s are the mean of at least three separate experiments. Lower and upper 95% confidence limits of EC_50_s and IC_50_s are indicated. Maximal activities (% max act) of the chemicals tested for their agonistic activity and minimal activities (% min act) of the chemicals tested for their antagonistic activity are expressed as a percentage of the maximal luciferase activity induced by 10^−8^ M aldosterone. They were determined at 10^−5^ M for aldosterone, canrenone, cortisol, dexamethasone, promegestone, mifepristone, and dihydrotestosterone. They were determined at 10^−6^ M for bimedrazole, spironolactone, and medrol, and at 10^−7^ M for drospirenone and 17α-hydroxy progesterone. Relative potency (REP) of each agonistic chemical was calculated as the ratio of concentrations of aldosterone or chemical required to induce the specific transactivation by 50% (ratio of EC50 values). REP value for aldosterone was arbitrarily set at 1. NA. Non-active.

Since luciferase expression was only induced by MR ligands in UG5LN-GAL4-hMR and UG5LN-GAL4-zfMR reporter cell lines, together with the use of UG5LN assay as a control for non-specific effect on luciferase, the established set of cell lines allows us to unambiguously characterize the potency and efficacy of natural and pharmaceutical steroids for hMR and zfMR.

### Characterization of the mineralocorticoid receptor activity of natural and synthetic steroids in UG5LN-GAL4-hMR and UG5LN-GAL4-zfMR cell lines

**Figure 4:**
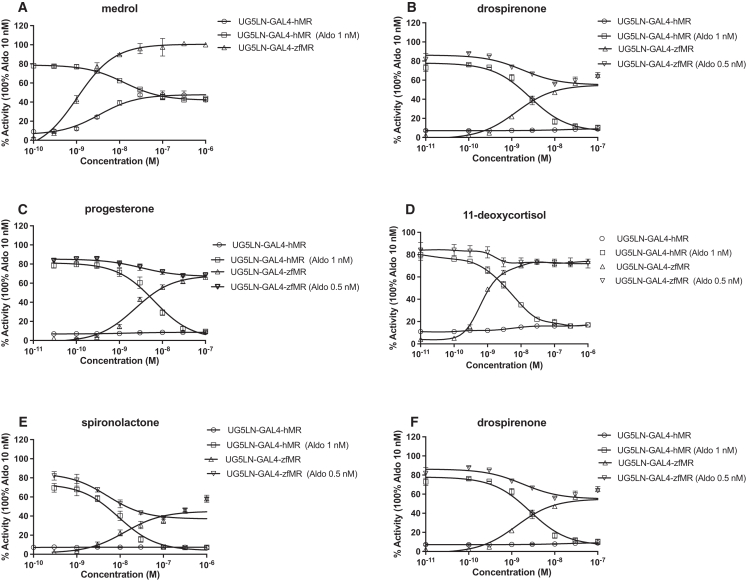
Medrol, drospirenone, progesterone, 11-deoxycortisol, spironolactone and drospirenone had an higher efficacy on zfMR than on hMR Dose-response curves of medrol (A), drospirenone (B), progesterone (C), 11-deoxycortisol (D), spironolactone (E), and drospirenone (F) in UG5LN-GAL4-hMR and UG5LN-GAL4-zfMR cells. Chemicals were tested alone (agonism test) or in the presence of aldosterone 1 nM in UG5LN-GAL4-hMR cells and 0.5 10^−9^ M in UG5LN-GAL4-zfMR cells (antagonism test). Results are expressed as the percentage of the maximum luciferase activity induced by 10^−8^ M aldosterone. The curves are presented as a non-linear regression; log (ligand) versus response. EC_50_ values (10^−9^ M) and IC_50_ values (10^−9^ M) are shown in Tables 3 and 4. Data are presented as means ± SD values. n (replicates per experiment) = 4 per group.

In order to further investigate the species differences between hMR and zfMR, we tested other steroids in the two reporter cell lines. The synthetic glucocorticoid medrol, used as an anti-inflammatory drug, and the natural progestogen 17α-hydroxyprogesterone showed differences in efficacy, being partial agonists on hMR and full agonists on zfMR (Figures 4A and 4B). The two chemicals were also slightly more potent on zfMR than on hMR. Indeed, the EC_50_s of Medrol and 17α-hydroxyprogesterone are 4.1 and 0.9 10^−9^ M for hMR and 1.8 and 0.2 10^−9^ M for zfMR, respectively (Tables 3 and 4). Because these two compounds only partially induced hMR, their activity toward this receptor was further characterized after co-exposure of UG5LN-GAL4-hMR cells to these two chemicals in the presence of aldosterone 10^−9^ M to assess their antagonistic activity on hMR. Medrol and 17α-hydroxyprogesterone were able to inhibit aldosterone-induced luciferase in a concentration manner down to the level of activity obtained with the compound alone, which represents the typical response profile of a partial agonistic ligand. The calculated IC_50_ values are 12.9 and 4 10^−9^ M for medrol, and 17α-hydroxyprogesterone, respectively (Table 3).

The natural progestogen progesterone, the natural glucocorticoid 11-deoxycortisol, the pharmaceutical anti-mineralocorticoid spironolactone, and the synthetic progestogen drospirenone also showed differences in efficacy as they were all able to partially activate the zfMR but not the hMR, toward which they were instead complete antagonists (Figures 4C, 4D, 4E, and 4F, respectively). Spironolactone and progesterone had already been reported to be partial agonists of the zfMR and full antagonists of the hMR,^16^^,^^23^ whereas regarding 11-deoxycortisol and drospirenone, to the best of our knowledge, no other studies have investigated interactions with the MR of other species than humans. The EC_50_ values of progesterone, 11-deoxycortisol, spironolactone, and drospirenone for zfMR are 2.3, 0.73, 7.3, and 1.7 10^−9^ M, respectively. The four chemicals were able to inhibit aldosterone-induced luciferase activity in the two MR reporter cells with the typical response profile of a partial agonistic ligand for zfMR and full antagonist for hMR. The calculated spironolactone and drospirenone IC_50_ values are 5.6 and 2.1 10^−9^ M for zfMR. For hMR, the calculated progesterone, 11-deoxycortisol, spironolactone, and drospirenone IC_50_ values are 6.4, 4.4, 9.9, and 2.7 10^−9^ M for hMR, respectively (Tables 3 and 4).

**Figure 5:**
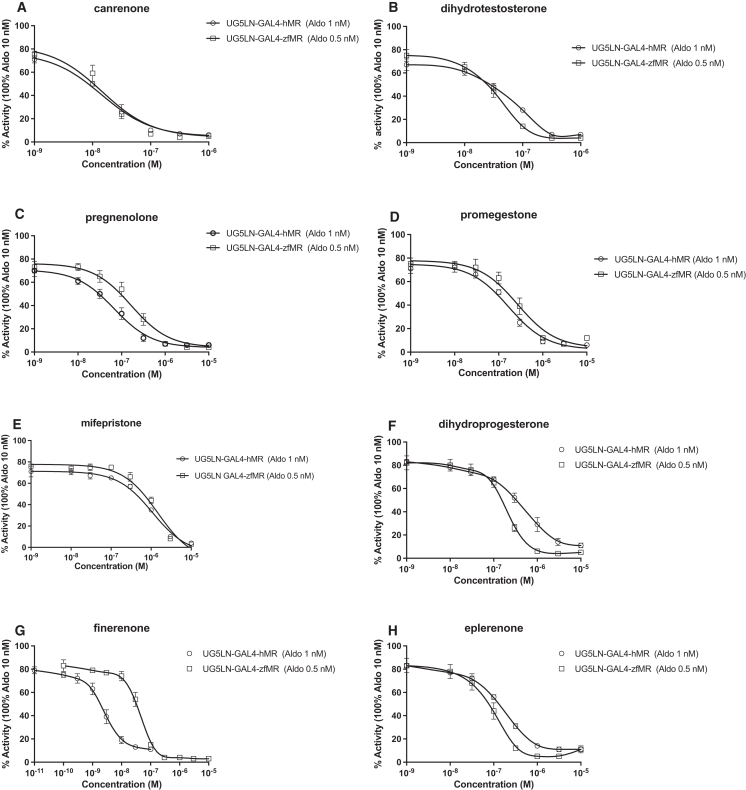
Canrenone, dihydrotestosterone, pregnenolone,promegestone, mifepristone dihydroprogesterone, finerenone and eperone fully antagonized hMR and zfMR (A–H) Dose-response curves of canrenone (A), dihydrotestosterone (B), pregnenolone (C), promegestone (D) mifepristone (E), dihydroprogesterone (F), finerenone (G), and eplerenone (H) in UG5LN-GAL4-hMR and UG5LN-GAL4-zfMR cells. Chemicals were tested in the presence of aldosterone 10^−9^ M in UG5LN-GAL4-hMR cells and 0.5 10^−9^ M in UG5LN-GAL4-zfMR cells (antagonism test). Results are expressed as the percentage of the maximum luciferase activity induced by 10^−8^ M aldosterone. The curves are presented as a non-linear regression; log (ligand) versus response. IC_50_ values (10^−9^ M) are shown in Tables 3 and 4. Data are presented as means ± SD values. n (replicates per experiment) = 4 per group.

Finally, the antimineralocorticoids canrenone, finerenone, and eplerenone, the androgen dihydrotestosterone, the progesterone-precursor pregnenolone, the progestins promegestone and dihydroprogesterone, and the antiprogestin mifepristone were all full antagonists on both hMR and zfMR (Figures 5A–5H, respectively). The calculated canrenone, dihydrotestosterone, pregnenolone, promegestone, mifepristone, dihydroprogesterone, finerenone and eplerenone IC_50_ values are 18, 35, 166, 291, 1055, 186, 40 and 88 10^−9^ M for zfMR and 17, 60, 68, 169, 836, 395, 2.4 and 152 10^−9^ M for hMR, respectively (Tables 3 and 4). The IC_50_ values for hMR and zfMR of the different chemicals are slightly different except for finenerone, which seems to be more selective for hMR than for zfMR.

### Characterization of the mineralocorticoid receptor activity of natural and pharmaceutical steroids in UG5LN-GAL4-hMR (T870L) cell line

**Figure 6:**
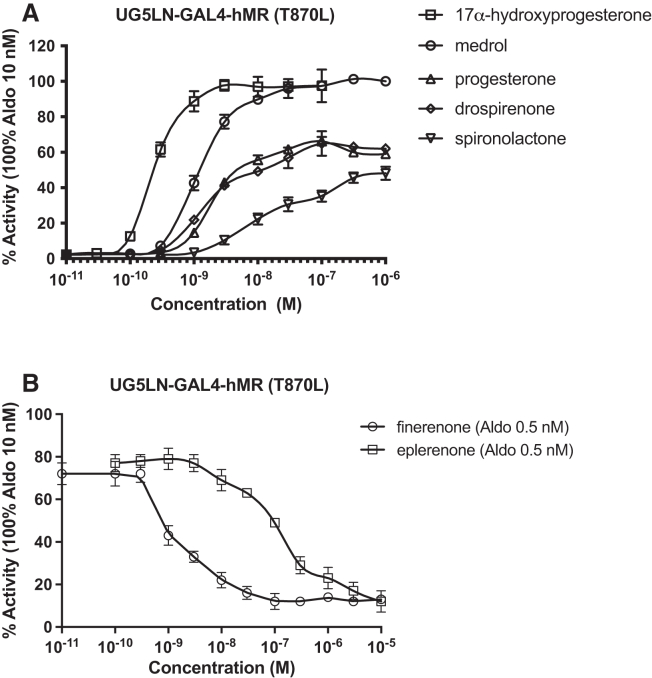
The substitution of the threonine 870 by a leucine increased the efficacy of steroids on hMR Dose-response curves of (A) 17α-hydroxyprogesterone, medrol, progesterone, drospirenone, and spironolactone and (B) finerenone and eplerenone in UG5LN-GAL4-hMR (T870L) cells. 17α-hydroxyprogesterone, medrol, progesterone, drospirenone, and spironolactone were tested alone (agonism test). (B) Finerenone and eplerenone were tested in the presence of aldosterone 0.5 10^−9^ M (antagonism test). Results are expressed as the percentage of the maximum luciferase activity induced by 10^−8^ M aldosterone. The curves are presented as a non-linear regression; log (ligand) versus response. EC_50_ values (10^−9^ M) and IC_50_ values (10^−9^ M) are shown in Tables 5 and 6. Data are presented as means ± SD values. n (replicates per experiment) = 4 per group.

The interspecies differences of efficacy observed in this study with spironolactone and progesterone have already been reported by Fuller et al.^16^ and could be explained by the substitution of a threonine by leucine in helix 8 of the hMR ligand-binding domain, which confers the agonist response of these 2 chemicals. To determine if the differences of efficacy observed with the other steroids medrol, 17α-hydroxyprogesterone, and drospirenone were also due to this substitution, we established a reporter cell line (UG5LN-GAL4-hMR (T870L), in which threonine in position 870 of hMR is mutated to leucine. We then tested these steroids, including progesterone and spironolactone (Figure 6A). Medrol and 17α-hydroxyprogesterone, which are partial agonists in the UG5LN-hMR cells, are full agonists in the UG5LN-hMR (T870L) cells (Table 5) as in UG5LN-zfMR. Progesterone, drospirenone, and spironolactone, which are full antagonists in the cells expressing hMR, are partial agonists in the cells expressing the mutated hMR (maximal activity of 66, 65, and 48%, Table 5) or zfMR. Thus, in these cells expressing the mutated hMR, the efficacy of hMR transactivation by the five chemicals was significantly enhanced, which confirms that the differences in efficacy observed for all these steroids are mainly due to this single amino acid difference.

**Table 5:** Agonistic hMR (T870L) activity assessment

Ligands	EC50 (10^−9^M)	lower and upper 95% conf. limit (10^−9^M)	% max act
DMSO	–	–	2.2 ± 0.2
17α-hydroxyprogesterone	2.5	2.1 to 3	97.6 ± 5.6
medrol	1.3	1.1 to 1.8	101.3 ± 5.3
progesterone	1.9	1.6 to 2.4	66.2
drospirenone	1.7	1.2 to 2.6	64.9
spironolactone	18.8	11 to 28	48.1

Half maximal effective concentration (EC_50_) and maximal activity (% max act) of the agonistic chemicals on hMR (T870L).

EC_50_s are expressed in nanomolar (10^−9^ M). Values of EC_50_ are the mean from at least three separate experiments. Lower and upper 95% confidence limits of EC_50_s are indicated. Maximal activities (% max act) of the chemicals tested at 10^−6^ M for their agonistic activity are expressed as a percentage of the maximal luciferase activity induced by 10^−8^ M aldosterone.

As we observed that finerenone was a more potent antagonist on hMR than zfMR (Figure 5F; Tables 4 and 5), we also tested it in the UG5LN-GAL4-hMR (T870L) reporter cell line to investigate if the mutation influences the affinity of the chemical for the receptor. In these cells, finerenone behaves as a full antagonist with an IC50 of 1.4 10^−9^ M, which is similar to the IC50 of hMR (Figure 6B; Table 6). This indicates that the difference in potency is probably due to another amino acid difference between hMR and zfMR which needs to be further identified.

**Table 6:** Antagonistic hMR (T870L) activity assessment

Ligands	IC50 (10^−9^M)	lower and upper 95% conf. limit (10^−9^M)	% max act
finerenone	1.41	0.91 to 2.17	13 ± 1.6
eplerenone	114	89 to 145	12 ± 2.5

Half maximal inhibitory concentration (IC_50_) and minimal activity of the antagonistic chemicals on hMR (T870L).

IC_50_s are expressed in nanomolar (10^−9^ M). Values and IC_50_s are the mean from at least three separate experiments. Lower and upper 95% confidence limits of IC_50_s are indicated. Minimal activities (% min act) of the chemicals tested at 10^−5^ M for their antagonistic activity are expressed as a percentage of the maximal luciferase activity induced by 10^−8^ M aldosterone.

### Characterization of the mineralocorticoid receptor activity of environmental chemicals in UG5LN-GAL4-hMR and UG5LN-GAL4-zfMR cell lines

**Figure 7:**
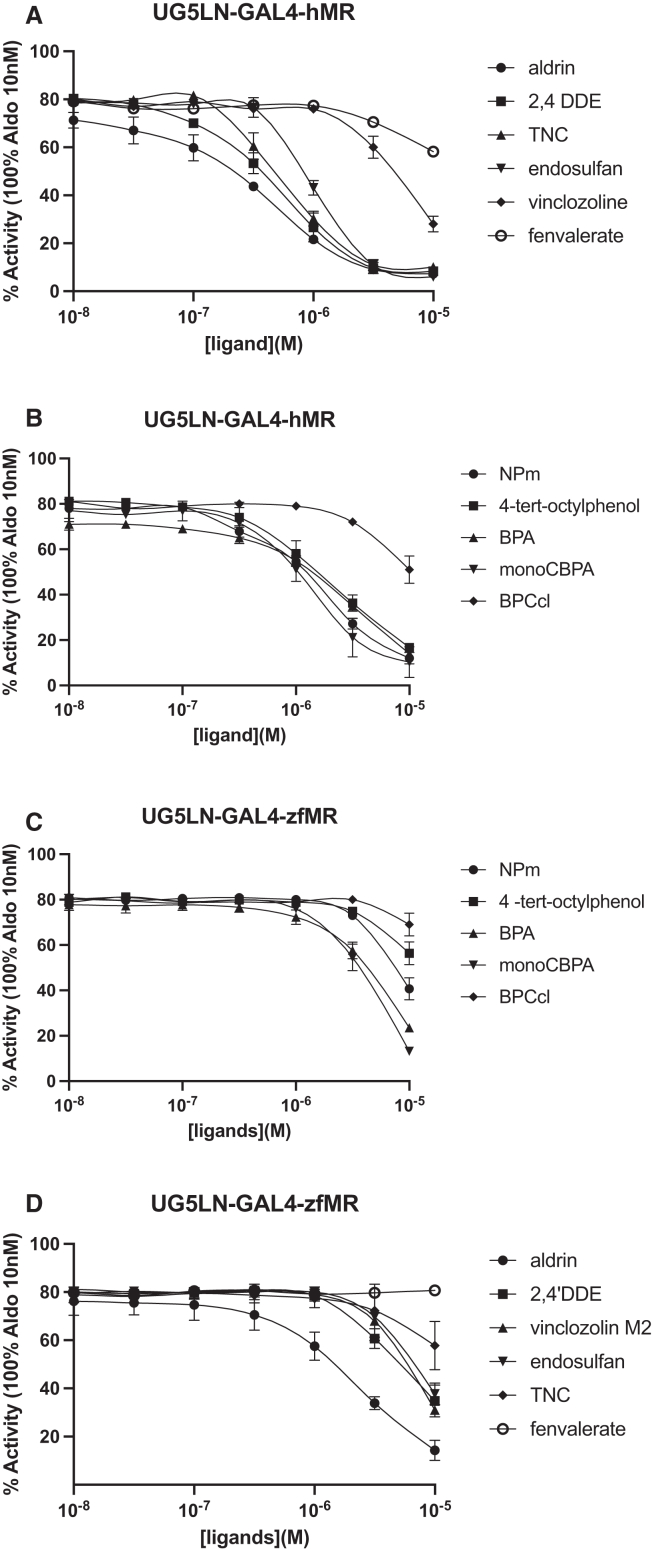
HMR and zfMR are targets of environmental chemicals Dose-response curves of environmental chemicals in UG5LN-GAL4-hMR (A and B) and UG5LN-GAL4-zfMR (C and D) cells. Chemicals were tested in the presence of aldosterone 10^−9^ M in UG5LN-GAL4-hMR cells and 0.5 10^−9^ M in UG5LN-GAL4-zfMR cells (antagonism test). Results are expressed as the percentage of the maximum luciferase activity induced by 10^−8^ M aldosterone. The curves are presented as a non-linear regression; log (ligand) versus response. IC_50_ values (10^−6^ M) are shown in Table 7. Data are presented as means ± SD values. n (replicates per experiment) = 4 per group.

It is well known that environmental chemical contaminants, such as pesticides and other industrial compounds used in everyday activities, can target the NRs, such as ER and AR. To investigate whether also hMR and zfMR are similarly targeted by these substances, we tested 13 ECs known for their anti-androgenic activity^5^^,^^6^^,^^24^^,^^25^^,^^26^ in the two established cell lines UG5LN-GAL4-hMR and UG5LN-GAL4-zfMR (Figures 7A–7D). These included industrial compounds (nonylphenol mixture, 4-*tert*-octylphenol, bisphenol-A, 2-chloro-bisphenol A, bisphenol C), pesticides (aldrin, chlordecone, endosulfan, HPTE, transnonachlor, 2,4′DDE), and pesticide metabolites (HPTE and vinclozolin M2) (Table 2). None of these environmental chemicals induced the expression of luciferase activity when tested alone as potential agonists (data non shown). Conversely, they showed antagonistic activity toward hMR and zfMR (Figures 7A–7D), confirming that MR is a target of environmental chemicals, as is AR. The most active chemicals on hMR were aldrin, 2,4′DDE, and transnonachlor with IC_50_s of 0.39, 0.47, and 0.61 10^−6^ M on hMR (Figure 7B; Table 7). The most active chemicals on zfMR were aldrin and transnonachlor with IC_50_ of 2.17 and 2 10^−6^ M (Figure 7D; Table 7). Bisphenol C and vinclozolin M2 displayed similar potency for hMR and zfMR (Figures 7A–7D; Table 7). The other chemicals were more potent antagonists of hMR than zfMR. Overall, the environmental chemicals tested showed antagonistic activities with minor differences between hMR and zfMR with regard to active concentrations. It is noteworthy that they exerted their anti-MR activity with much lower potencies than those exerted by the steroids (micromolar versus nanomolar range), which is in agreement with published studies for pesticides and hMR.^8^^,^^27^

**Table 7:** Antagonistic activity hMR and zfMR assessment

Ligands	UG5LN-GAL4-hMR			UG5LN-GAL4-zfMR		
	IC50 (10^−6^M	lower and upper 95% conf. limit (10^−6^M)	% min act	IC50 (10^−6^M)	lower and upper 95% conf. limit (10^−6^M)	% min act
nonylphenol mixture	1.56	1.32 to 1.84	12 ± 2.5	9.20	8.92 to 9.50	40.7 ± 4.8
4-*tert*-octylphenol	2.12	1.91 to 2.36	16.7 ± 1.9	15.80	13.7 to 19.0	56.4 ± 5
bisphenol A	2.28	1.38 to 3.57	15 ± 2.4	5.15	4.82 to 5.49	24.0 ± 2.6
2-chloro-bisphenol A	1.36	1.13 to 1.65	12 ± 8.7	4.30	3.80 to 4.30	13.2 ± 1.3
bisphenol C	13.2	11.7 to 15.6	51 ± 6	11.8	10.0 to 13.2	69,0 ± 5
2,4′-DDE	0.47	3.77 to 5.76	8.3 ± 1.2	6.90	5.98 to 8.0	34.8 ± 6.6
vinclozoline M2	5.96	5.34 to 6.66	28 ± 3.3	7.0	6.62 to 7.39	31 ± 0.5
chlordecone	1.21	1.08 to 1.36	8.4 ± 1	5.59	5.39 to 5.79	28.6 ± 0.3
transnonachlore	0.61	0.53 to 0.70	10 ± 0.9	2,0	1.76 to 2.35	57.8 ± 9
endosulfan	1.01	0.96 to 1.06	6 ± 1	8.40	8.0 to 8.82	37.7 ± 4.5
aldrin	0.39	0.25 to 0.46	7 ± 2.5	2.17	2.05 to 2.29	17.3 ± 3.7
pretilachlore	3.51	1.91 to 6.04	21.5 ± 1.7	4.71	4.32 to 5.14	21 ± 0.7
HPTE	2.06	1.64 to 2.56	12.3 ± 1.5	11.1	10.0 to 12.0	51 ± 6.8

Half maximal inhibitory concentration (IC_50_) and minimal activity of the environmental chemicals on hMR and zfMR.

IC_50_s are expressed in microolar (10^−6^ M). Values of IC_50_ are the mean from at least three separate experiments. Lower and upper 95% confidence limits of IC_50_s are indicated. Minimal activities (% min act) of the chemicals tested at 10^−5^ M for their antagonistic activity are expressed as a percentage of the maximal luciferase activity induced by 10^−8^ M aldosterone.

### Detection of mineralocorticoid receptor activities in environmental water samples using UG5LN-GAL4-MR cell lines

As we have newly found that a large range of environmental contaminants can potentially interfere with MR, either as agonists and/or antagonist, we were interested in assessing the relevance of the newly established cell lines to detect the presence of MR active substances in real environmental samples. For this purpose, we selected urban WWs, a contaminated environmental compartment that is contaminated by and representative of the chemical discharges from many anthropogenic activities. Furthermore, urban WWs are a well-identified source of endocrine active chemicals in the aquatic environment.^28^^,^^29^

**Figure 8:**
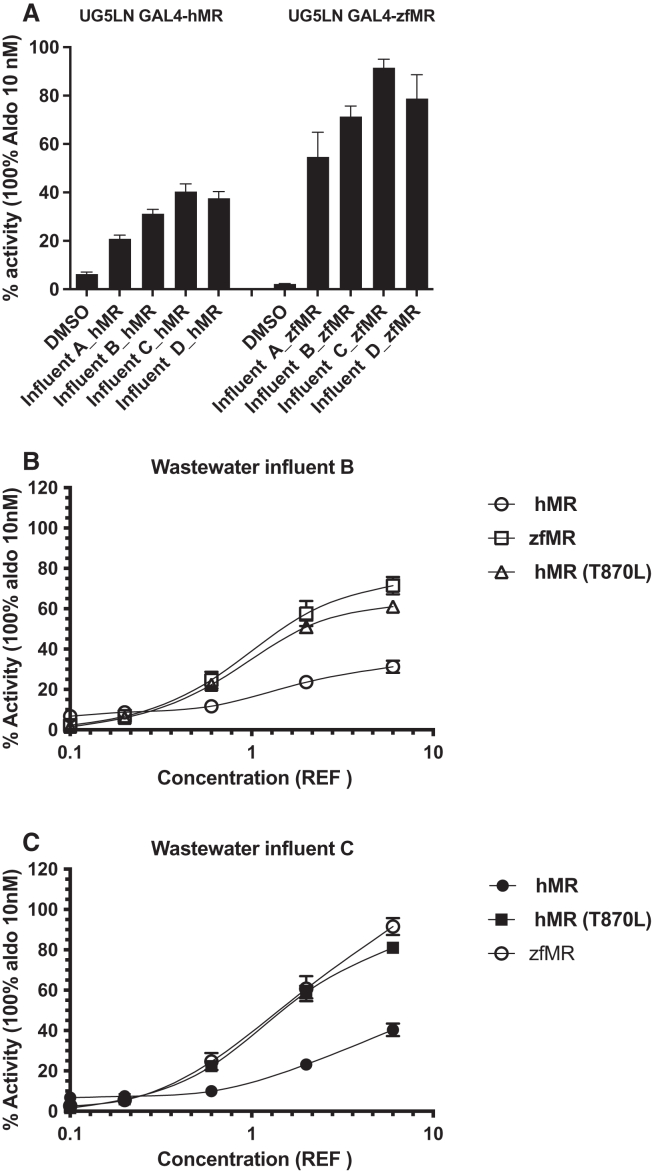
hMR and zfMR activities of waste water samples (A–C) Mineralocorticoid activities of waste water influent and effluent of two urban sites, A and B, were tested in the UG5LN GAL4-hMR and UG5LN-GAL4-zfMR cells. Firstly, influent and effluent of the two sites were tested at the maximal concentration of 3 REF for influents and 6 REF for effluents (A). Secondly, serial dilutions of waste water influents A (B) and B (C) were tested in UG5LN-GAL4-hMR, UG5LN-GAL4-zfMR, and UG5LN-GAL4-hMR (T870L) cells. Sample concentration data are expressed in REF. Results are expressed as the percentage of the maximum luciferase activity induced by 10^−8^ M aldosterone. The curves are presented as a non-linear regression; log (REF) versus response. Data are presented as means ± SD values. n (replicates per experiment) = 4 per group.

Organic extracts of WWTP influents and effluents from two urban sites were tested on MR cell lines. Wastewater effluents showed either no (hMR) or low (zfMR) mineralocorticoid activity, as compared to the solvent control. The WW influents exerted higher agonistic activity, with higher efficacy on zfMR than hMR (Figure 8A). This agonistic mineralocorticoid activity was concentration-dependent (Figures 8B and 8C). Interestingly, a similar stronger efficacy was observed for zfMR and mutant hMR (T870L) (Figures 8B and 8C), confirming that the mutated amino acid in the ligand binding domain confers the change of activity, as observed with several steroid compounds (Figure 6A). Further chemical analysis will be necessary to identify the nature of the chemicals present in these extracts, but it is likely that the MR activity was mediated by one or several steroids with partial agonistic activity on hMR and higher efficacy for zfMR than for hMR. For instance, dexamethasone, medrol, and 17α-hydroxy-progesterone, which exert such an hMR/zfMR agonistic profile, are potential candidate chemicals to be investigated using target or suspect screening analyses to explain the differences in hMR and zfMR activities. Effect-directed analysis (EDA) would further characterize the MR activities of the samples by isolating the bioactive fractions on both receptors and thus facilitate the structural identification of the responsible chemicals.^30^ Our future work on this type of active samples will therefore involve isolating the hMR and zfMR active fractions and identifying the active chemicals in these fractions with the aid of chemical analysis using mass spectrometry.

## Discussion

The UG5LN-MR cell lines have proved to be a useful tool to characterize human and zebrafish differences in MR activation or inhibition by several chemicals. Species-specific differences in MR activation had already been reported showing a divergent evolution of MR between terrestrial vertebrates and fish.^15^^,^^16^^,^^31^^,^^32^ In our study, we observed that aldosterone activates hMR and zfMR with slightly better potency for the latter. This hormone is the natural ligand of hMR in epithelial tissues (such as the distal collector tubules of the kidney and colon) and is recognized as a tetrapod-specific hormone.^33^ In fish, aldosterone is not produced, and emerging evidence suggests that cortisol could be the primary corticosteroid in these species, mediating a plethora of physiological parameters, including the hydromineral balance.^34^^,^^35^ Accordingly, we reported that cortisol has slightly more efficacy and potency on zfMR than on hMR. We also observed that progesterone is an agonist of zfMR, while completely antagonizing hMR. This is in line with the literature, as this steroid has been reported to be a potent activator of MR in trout, sturgeon, gar, and zebrafish.^16^^,^^31^^,^^36^^,^^37^ Interestingly, progesterone has also been proposed as a candidate for a physiological activator of zfMR^36^^,^^38^ since in mammals it is the physiological activator of PR, while in fish this role is played by 17α,20β-dihydroxyprogesterone (DHP).^39^ Therefore, it has been suggested that progesterone could be the physiological activator of MR in fish.^15^ 17α-hydroxyprogesterone is a partial agonist on hMR and a full agonist on zfMR, indicating that this hormone may also play an important role in regulating MR in fish. Finally, 11-deoxycortisol is an agonist of zfMR, while completely antagonizing the hMR.

We observed that spironolactone and drospirenone were partial agonists of zfMR, whereas they completely antagonized the hMR. The ability of progesterone and spironolactone to activate zfMR has already been reported.^16^^,^^23^^,^^38^ This activity has been attributed to the substitution of a leucine (zfMR) by a threonine (hMR) in helix 8 of the ligand-binding domain of the receptor. This leucine interacts with a leucine located in helix 1 of the LBD, and this interaction results in the stabilization of the agonist conformation, which includes a strong interaction between helices 3 and 5.^16^ This stabilization of the agonist conformation in the absence of ligand can induce a decrease in affinity for co-repressors or an increase in affinity for co-activators. Thus, compounds antagonistic to hMR become partial agonists for zfMR (spironolactone and drospirenone) and partial agonists for hMR become full agonists for zfMR (medrol, 17α-hydroxyprogesterone, 11-deoxycortisol).

Interestingly, we reported that dexamethasone and medrol are partial agonists on hMR while they are full agonists on zfMR. Our experiments with the UG5LN-GAL4-hMR (T870L) reporter cell line seem to indicate that the difference of efficacy observed for several steroids between hMR and zfMR is due to the single amino acid substitution. Interestingly, this leucine is conserved in several fishes (rainbow trout, tropical gar, medaka, sturgeon, elephant shark, little skate, and others),^16^^,^^39^ indicating that the results that we have obtained on zfMR could be extrapolated to other fishes. This evolution of fish MRs (threonine being replaced by leucine) makes them more inclined to recruit co-activators instead of co-repressors and be activated by different steroids, and is very probably linked to the fact that fish do not synthesize aldosterone, so they have adapted to be activated by other endogenous steroids.

Regarding the environmental chemicals tested on the two established UG5LN-GAL4-hMR and UG5LN-GAL4-zfMR cell lines, they all showed antagonistic activity on both hMR and zfMR. Aldrin, transnonachlor, and 2,4′DDE, are the most potent on hMR. Interestingly, these chemicals are more potent on hMR than bisphenol C and vinclozolin M2, which were identified as the most potent antiandrogens among ECs.^3^^,^^6^^,^^25^^,^^26^ In a similar way, most of the ECs tested were more potent on hMR than on zfMR, indicating that a larger screening of chemicals would improve the understanding of this species-specific difference.

Finally, the organic extracts of urban WWs tested on the cell lines UG5LN-GAL4-MR cell lines showed the presence of MR active chemicals in these samples, with higher activity on zfMR than on hMR. These specific profiles suggested that the main active chemicals in these samples could be steroids with higher efficacy on zfMR than on hMR, such as dexamethasone, medrol, 17α-hydroxy-progesterone, or 11-deoxycortisol. These pharmaceuticals, among many others, are plausible candidates as they are known to occur in such urban WW samples, originating from either domestic, hospital, or industrial activities.^29^^,^^40^ It is also not excluded that the resulting activity of the samples was due to a mixture of chemicals which part of which have more efficacy on zfMR than on hMR. It would be very interesting to test a larger number of environmental samples subjected to different types of pollution on these cells, such as urban, industrial, and agricultural on these cells to investigate putative differences in the effects. It is possible that urban sites show agonist activity (with higher potency on zfMR than on hMR), while industrial or agricultural sites may instead show antagonist activity (with similar potency for zfMR and hMR). On these samples, effect-directed analysis (EDA) would help further characterize the MR activities of the samples by isolating the bioactive fractions on both receptors and thus facilitating the structural identification of the responsible chemicals using high resolution chemical analysis by mass spectrometry.^30^ This would provide a better understanding of the nature of MR ligand environmental contamination depending on the type of site, and to determine whether MR activities are agonist or antagonist, and/or whether they differ between species. Finally, investigating the *in vivo* effects on zebrafish exposed to the different identified active chemicals, especially at early developmental stages, will provide valuable information on the toxicological significance of the zfMR-specific activity observed *in vitro*. Based on our results, which will have to be demonstrated on a larger number of samples, it is likely that fish from sites exposed to discharges from urban sites may show effects due to exposure to agonist mineralocorticoid molecules. On the other hand, we would expect fish at sites exposed to purely agricultural or industrial discharges to show effects due to exposure to antagonistic mineralocorticoid molecules. Our future work will therefore involve studying a larger number of sites for their hMR and zfMR activities, identifying the products active at these sites, and, if possible, investigating their possible effects on fish.

### Limitations of the study

Our study consisted of a preliminary investigation of differences in activation by chemicals of hMR and zfMR using reporter cell lines. It would be interesting to confirm the different activities of chemicals (antagonists, partial agonists, full agonists) on the expression of endogenous mineralocorticoid regulated genes in hMR positive cells. Similarly, experiments conducted on zebrafish (or other fish) would allow the activities of chemicals observed *in vitro* to be confirmed *in vivo* on zfMR. Finally, our study only looked at a limited number of environmental water samples. It would be interesting to test more samples of different types (water, soil, air) and exposed to different types of contamination (agricultural, industrial, and urban). In addition, it would be interesting to carry out chemical analyses to identify the active substances present in these samples.

## Resource availability

### Lead contact

Further information and requests for resources or reagents should be directed to and will be fulfilled by the lead contact Patrick Balaguer (patrick.balaguer@inserm.fr).

### Materials availability

All the plasmids used in this study are available under request.

HMLN hGR and UG5LN-GAL4-hMR cells are currently validated by the platform PEPPER (https://ed-pepper.eu). At the end of the validation (planned in September 2026), cells will be available under MTA with INSERM.

### Data and code availability

All data reported in this article as any additional information required to analyze the data reported in this article, will be shared by the lead contact upon request.

## Acknowledgments

This work was funded in part by the French projects 10.13039/501100007546ANSES TOXCHEM (2018/1/020), 10.13039/501100007546ANSES INDEE (2019-237), and by 10.13039/100018693Horizon Europe, the European Union’s 2021–2027 framework program for the funding of research and innovation under grant agreement No. 101057014 (project 10.13039/100006672PARC).

## Author contributions

Conceptualization and investigation: AT and PB; methodology: AT, MG, AB, AS, AE, YK, MB, and SAA; formal analysis and data curation: AT and PB; supervision: PB; funding acquisition: PB; writing – original draft: AT, AE, CG, and PB; visualization: AT and PB; writing – review and editing: AT, YK, MB, SAA, and PB.

## Declaration of interests

The authors declare no competing interests.

## STAR★Methods

### Key resources table

**Table:** 

REAGENT or RESOURCE	SOURCE	IDENTIFIER
**Chemicals and antibiotics**		

Puromycin	Sigma-Aldrich	P7255
Geneticin	Promega	V7983
Hygromycin	Sigma-Aldrich	SBR00308
Luciferin	Promega	E1605
Aldosterone	Sigma-Aldrich	A9477
Drospirenone	Sigma-Aldrich	SML0147
Spironolactone	Sigma-Aldrich	PHR2886
Canrenone	Sigma-Aldrich	SML1497
Finerenone	Sigma-Aldrich	TA9H97BAEA67
Eplerenone	Sigma-Aldrich	PHR3815
Bimedrazole	SANOFI	Gift
Cortisol	Sigma-Aldrich	NIST921A
Dexamethasone	Sigma-Aldrich	PR1526
Medrol	Sigma-Aldrich	PHR1717
11-deoxycortisol	LGC Standards	TRC-D232600-5 MG
Progesterone	Sigma-Aldrich	46665
Dihydroprogesterone	Sigma-Aldrich	P6285
Promegestone	Sigma-Aldrich	AABH97D36FE4
17α-hydroxyprogesterone	Sigma-Aldrich	H5752
Pregnenolone	Sigma-Aldrich	PHR2564
Mifepristone	Sigma-Aldrich	475838
Dihydrotestosterone	LGC Standards	DRE-C10255010
Nonylphenol mixture	Sigma-Aldrich	46018
4-*tert*-octylphenol	Sigma-Aldrich	290823
Bisphenol A	Sigma-Aldrich	239658
2-chloro Bisphenol A	Artmolecule	MR8046
Bisphenol C	Sigma-Aldrich	ATEH9A2C2D1B
Aldrin	Sigma-Aldrich	36666
Chlordecone	Sigma-Aldrich	45379
Endosulfan	Sigma-Aldrich	32015
Transnonachlore	Sigma-Aldrich	442811
2,4′DDE	LGC Standards	DRE-C120140000
HPTE	Sigma-Aldrich	T340944-1G
Vinclozolin M2	Sigma-Aldrich	AABH97CD814C

**Plasmids**		

pSG5-puromycin	H Gronemeyer (France)	N/A
pSG5-GAL4(DBD) (M1-S147)-puromycin	H Gronemeyer (France)	N/A
pSG5-GAL4(DBD)-hMR(LBD)-puromycin	This study	N/A
pSG5-GAL4(DBD)-hMRT870L(LBD)-puromycin	This study	N/A
pSG5-GAL4(DBD)-zfMR(LBD)-puromycin	This study	N/A
(GAL4RE)_5_βGlob-Luc-SVNeo	This study	N/A

**Oligonucleotides**		

hMR (M1-K984) sequence	Eurofins Genomics	N/A
zfMR (M1-K970) sequence	Eurofins Genomics	N/A
T870L mutagenesis forward primer	Eurofins Genomics	N/A
T870L mutagenesis reverse primer	Eurofins Genomics	N/A
GAL4 DBD forward primer	Eurofins Genomics	N/A
GAL4 DBD reverse primer	Eurofins Genomics	N/A

**Experimental cell lines**		

HeLa S3	H Gronemeyer (France)	N/A
U20S	ATCC	ATCC-CRL-3455
HMLN-hGR	Toso et al.^20^	N/A
UG5LN-GAL4-hMR	This study	N/A
UG5LN-GAL4-hMRT870L	This study	N/A
UG5LN-GAL4-zfMR	This study	N/A

**Software and algorithms**		

GraphPad Prism version 10.5.0	GraphPad Software Inc	N/A

### Experimental model and subject details

#### Chemicals

Stock solutions of the chemicals were stored in dimethyl sulfoxide (DMSO) at −20°C and fresh solutions of the tested chemicals were prepared prior to the experiment in experimental medium. The final concentration of DMSO did not exceed the 0.2% (v/v) of the experimental medium. Table 1 describes all tested natural and synthetic steroids, which were chosen as known steroid receptor ligands, while Table 2 lists all tested xenobiotics (i.e., pesticides and industrial compounds) selected as common ECs and/or suspected endocrine disruptors.

#### Cell culture

Cell culture material was from Life Technologies (Cergy-Pontoise, France) except the 96-well cell star plates, which were from Greiner Labortechnic (Poitiers, France).

### Method details

#### Plasmid construction

pSG5-puromycin and pSG5-GAL4(DBD) (M1-S147)-puromycin are kind gifts of H Gronemeyer (IGBMC, Illkirch, France). hMR (M1-K984) and zfMR (M1-K970) sequences were ordered to Eurofins Genomics (Ebersberg, Germany). To construct GAL4(DBD)-MR(LBD) fusion proteins, hMR(LBD) (G671-K894) and zfMR(LBD) (L668-K970) have been amplified by PCR and introduced between XhoI and Kpn I sites of pSG5-GAL4(DBD) (M1-S147)-puromycin. pSG5-GAL4(DBD)-hMRT870L(LBD) was obtained by site-directed mutagenesis based on PCR of GAL4(DBD)-hMR(LBD) (5′ primer GCTCACCTTTGAAGAATAC**CTG**ATCATGAAAGTTTTGC and 3′ primer GCAAAACTTTCATGAT**CAG**GTATTCTTCAAAGGTGAGC).

(GAL4RE)_5_βGlob-Luc-SVNeo was already described.^41^ Briefly, (GAL4RE)_5_βGlob-Luc-SVNeo contains a luciferase gene driven by five of yeast activator GAL4 binding sites in front of βglobin promoter and a neomycin phosphotransferase gene under the control of SV40 promoter, respectively. All the plasmids were verified by sequencing (Eurofins) and transient transfections.

#### Wastewater samples preparation

Wastewater samples were collected in July 2019 from urban wastewater treatment plants (WWTPs) with a treatment capacity of 120,000 (site A) and 40,000 (site B) population equivalents. Site A receives wastewater from domestic, industrial and hospital sources, while site B only receives wastewater from domestic and industrial sources. All samples were collected in solvent washed 1 L amber glass bottles. Wastewater influent (0.5L) and treated effluent (1 L) were collected in high density polyethylene bottles (Nalgène), transferred to the laboratory at 4°C where they were filtered on glass fiber filters (GF/F grade, Whatmann) and then stored at −20°C. Upon thawing, solid-phase extraction (SPE) was used to extract the dissolved organic phase. In brief, HLB columns (6cc, 200 mg, OASIS-HLB, Waters) were conditioned with 5 mL dichloromethane (DCM, Milipore, 99.8%), 5 mL of a mix v/v DCM/Methanol (MeOH, VWR chemicals, 99.9%), 5 mL MeOH then 5 mL of ultrapure water. After sample percolation, columns were dried for 2 h, then eluted with 10 mL of MeOH, 10 mL of a mix v/v MeOH/DCM and 10 mL of DCM. Extracts were evaporated to dryness (EZ-2plus, Genevac), and redissolved in 0.5 mL od DMSO for storage at −20°C. This sample preparation step allows a concentration of the dissolved organic phase by a factor of about 1000 (influent) to 2000 (effluent). To carry out the bioassays, the extracts were tested at the maximum concentration of 0.3% DMSO i.e., (3 REF for the influent and 6 REF for the effluent).

#### Cell culture and establishment of reporter gene cell lines

The U2OS cells used to establish the MR reporter cell lines were obtained from and verified by ECACC (UK Health Security Agency, Porton Down, Salisbury, UK). The strategy used has been described previously to establish other NR-based cell lines.^42^ It is summarized in Figure S1. First, the different pSG5-GAL4(DBD)-MRs (LBD) and (GAL4RE)_5_βGlob-Luc-SVNeo plasmids were tested by transient transfections in U2OS. Then, the UG5LN-GAL4-hMR and UG5LN-GAL4-zfMR clonal cells were generated in two steps. The first step was to establish clonal UG5LN cells by a stable transfection of U2OS cells, considered as steroid receptor negative, with the plasmid (GAL4RE)_5_βGlob-Luc-SVNeo. The second step consisted of the transfection of UG5LN cells with the plasmids pSG5-GAL4(DBD)-hMR(LBD)-puro, pSG5-GAL4(DBD)-hMRT870L(LBD)-puro or pSG5-GAL4(DBD)-zfMR(LBD)-puro, to generate UG5LN-GAL4-hMR, UG5LN-GAL4-hMRT870L and UG5LN-GAL4-zfMR clonal cells, respectively. Stable cell clones were selected in the presence of puromycin 0.5 μg/mL and G418 1 mg/mL. For each receptor, 5 to 10 clones were chosen for their ligand-induced luciferase expression. The clones were amplified and luciferase expression was checked at several passages. For each receptor, the clone with the best induction of luciferase activity was selected and used for the screening of the different chemicals. The stability and the inducibility of luciferase expression were checked during at least 20 passages (20 weeks). We also measured the expression of the different GAL4 fusion proteins by RT-PCR expression using GAL4-DBD specific primers (forward: 5′-AAGAAAAACCGAAGTGCGCC-3′, reverse: 5′-AGTCAGCGGAGACCTTTTGG-3′) to confirm that the different Gal4-MR fusion proteins were expressed at similar levels Expression of GAL4-hMR and GAL4-zfMR was found very similar, whereas that of GAL4-hMRT870L was slightly lower (data not shown).

Both the parental U2OS and stably transfected cell lines were cultured in Dulbecco’s Modified Eagle’s Medium: Nutrient Mixture F-12 (DMEM/F-12) containing phenol red and 1 g/L glucose and supplemented with 10% fetal bovine serum (FBS), 100 units/mL of penicillin and 100 μg/mL of streptomycin. The culture medium was supplemented with 1 mg/mL geneticin for UG5LN cells and 1 mg/mL geneticin and 0.5 μg/mL puromycin for UG5LN-GAL4-MR cells. Exposure to the test chemicals was carried out in phenol red-free DMEM experimental medium supplemented with 5% of dextran-coated charcoal FBS (DCC-FBS), 100 units/mL of penicillin and 100 μg/mL of streptomycin.

Both the parental U2OS and stably transfected cell lines were tested for mycoplasma contamination using the Lonza mycoplasma detection kit Mycoalert (Ozyme, Saint-Cyr-L’école, France).

#### Reporter gene assay

To perform the reporter gene assay, cells were seeded in 96-wells white transparent culture plates (Greiner bio-one 655098CellStar; Dutscher, Brumath, France) at a density of 60,000 cells per well in 150 μL of culture medium and incubated at 37°C and 5% CO2 for 24 h. Then, after the attachment period, medium was removed and cells were exposed to increasing dilutions of the tested compounds in the experimental medium (DMSO; final concentration 0.1% v/v for chemicals and 0.3% v/v for water sample extracts) and incubated at 37°C and 5% CO_2_. After 24 h, medium was removed and replaced with 50 μL/well of experimental medium containing 0.3 mM luciferin. The luminescence signal was monitored in intact live cells for 2 s per well using a MicroBeta Wallac luminometer (PerkinElmer, Courtaboeuf, France). All experiments were performed in quadruplicate.

The activities of the chemicals tested were expressed as a percentage of the maximal luciferase activity induced by aldosterone (10 nM) that was added as a positive control in each plate. In the antagonist mode, to assess the ability of the chemicals to inhibit aldosterone-induced MR transactivation, UG5LN-GAL4-hMR and UG5LN-GAL4-zfMR cells were co-exposed to the chemicals of interest in addition to aldosterone at concentration of 1 nM and 0.5 nM, respectively, concentrations that produce approximately 80% of its maximum luciferase response.

To ensure that the effect of the chemicals is due to a direct and specific interaction with the MR (and not to a general effect on transcription for instance), we also tested these products in UG5LN cells (i.e., with the luciferase gene but lacking MR). In these cells, we observed no non-specific modulation of luciferase expression by any of the tested chemicals or water extracts (data not shown). In addition, as a control for true antagonism, the effect of the chemicals exerting an antagonistic activity was tested in the presence of aldosterone at high concentration (1 μM) to verify that the inhibition observed with these compounds is competitive in nature.

#### MTT assay

The absence of effect on cell viability of the tested chemicals and environmental samples at the tested concentrations was assessed with the 3-(4,5-Dimethyl-2-thiazolyl)-2,5-diphenyl-2H-tetrazolium Bromide (MTT) assay. Briefly, after luminescence detection, medium containing luciferin was removed and replaced with 100 μL/well of test medium containing 0.4 mg/mL MTT for 4 h. Colorimetric signal was monitored at 570 nM using a Pherastar microplate reader (BMG Labtech, Champigny s/Marne, France). Tested chemicals or environmental samples must not exceed 10% mortality or they are considered to be cytotoxic and must be tested again at lower concentrations. We did not observed toxicity higher than 10% for the different natural, pharmaceutical and environmental chemicals at concentrations lower than 10 μM. Similarly, we did not observed toxicity for environmental samples at concentrations lower than 6 REF.

Graphical abstract was created with BioRender.com.

### Quantification and statistical analysis

The quantification and statistical analysis are integral parts of the software used (GraphPad Prism 10, GraphPad Software Inc). The compounds were tested at different concentrations in at least three independent experiments. In each experiment, the compounds were tested in quadruplicate for each concentration, and the data were expressed as averages of the quadruplicates with their standard deviation to obtain a single value for that experimental series. Then, individual dose-response curves for each chemical were fitted from the averages of the three individual experiments using the sigmoidal dose-response function of the graphics and statistics software program GraphPad. Specifically, GraphPad employs nonlinear regression with the least squares method to fit the data to a sigmoidal function (based on the Hill equation). This method is used to determine the optimal parameter values, such as the Half Maximal Effective concentration (EC_50_) for agonist and the Half Maximal Inhibitory Concentration (IC_50_) for antagonist. For a given agonist, the EC_50_, i.e., the concentration inducing 50% of its maximal effect, was calculated, while for an antagonist, the IC_50_, i.e., the concentration required for 50% inhibition, was calculated. Once the best-fit curve was obtained, the confidence intervals (e.g., 95% CI) for these parameters were estimated based on the uncertainty (variance) derived from the regression model, and the lower and upper 95% confidence limits of EC_50_s and IC_50_s were reported.

Water extracts were also tested in quadruplicate at various concentrations in at least three independent experiments. The expression of luminesce data and statistical analyses were performed in the same manner as for individual chemicals using GraphPad, with the only difference being that the concentration values were expressed in units of relative enrichment factor (REF). The REF accounts for sample enrichment by SPE and dilution in the bioassay.
