# Supplementary material for: Human and zebrafish mineralocorticoid receptors reporter cell assays to assess the activity of chemicals
Source: iScience. 2025 Oct 14;28(11):113764. doi: 10.1016/j.isci.2025.113764 (PMC12636382; doi:10.1016/j.isci.2025.113764)
Supplement: Document S1. Figure S1 [file mmc1.pdf]

iScience, Volume *28*

Supplemental information

Human and zebrafish mineralocorticoid receptors reporter cell assays to assess the activity of chemicals

Anna Toso, Abdelhay Boulahtouf, Marina Grimaldi, Audrey Sansaloni, Yoshinao Katsu, Michael E. Baker, Aurélie Escande, Clémentine Garoche, Selim Aït-Aïssa, and Patrick Balaguer

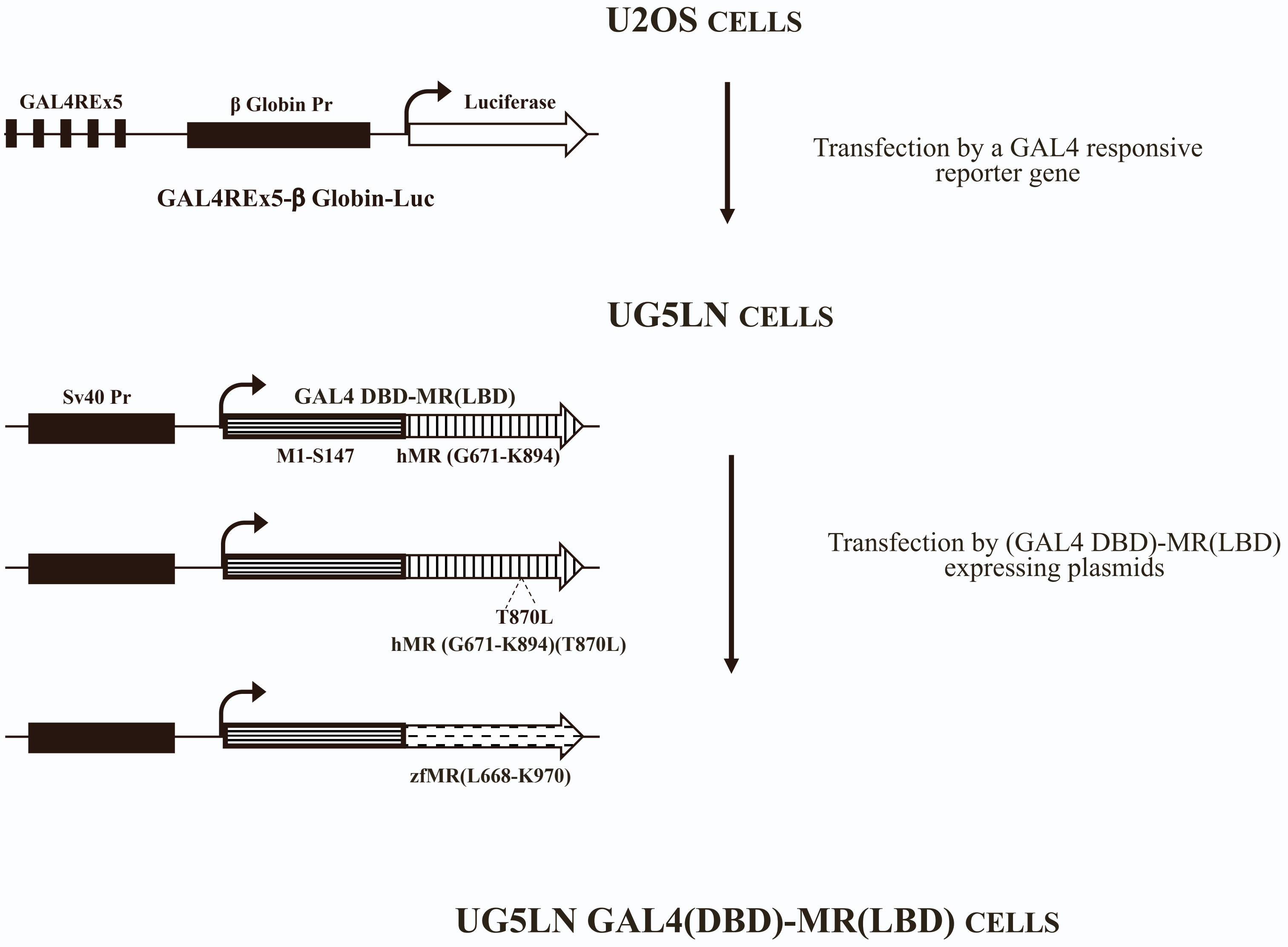

Supplementary Figure 1. Strategy of establisment of the different MR reporter cell lines.
